# T-cell exhaustion and antibody response in SARS-CoV-2 vaccine recipients

**DOI:** 10.17843/rpmesp.2025.424.14687

**Published:** 2025-12-01

**Authors:** Iván Lozada-Requena, Camila A. Arones-Santayana, Angela Vidal Riva, Joel de León, Arturo Pareja Cruz

**Affiliations:** 1 Immunology Laboratory, Department of Cellular and Molecular Sciences, School of Sciences and Engineering, Universidad Peruana Cayetano Heredia, Lima, Peru. Universidad Peruana Cayetano Heredia Immunology Laboratory, Department of Cellular and Molecular Sciences School of Sciences and Engineering Universidad Peruana Cayetano Heredia Lima Peru; 2 Virology Research Center, Universidad de San Martín de Porres, Lima, Peru. Universidad de San Martín de Porres Virology Research Center Universidad de San Martín de Porres Lima Peru

**Keywords:** Cellular Exhaustion, IgG, TNF-a, T Cells, PD-1, Vaccine

## Abstract

**Objectives.:**

To evaluate the percentage variations of the total lymphocyte population, T cells (TC), and their subpopulations; to characterize the cellular exhaustion profiles in CD4+ and CD8+ T cells; and to quantify anti-spike IgG antibody levels in individuals who received different vaccination schedules.

**Materials and methods.:**

We isolated PBMCs (peripheral blood mononuclear cells) from 45 vaccinated and 15 unvaccinated participants to measure TC percentages and MFI (mean fluorescence intensity) by flow cytometry. Additionally, we obtained serum from 42 vaccinated participants, 9 unvaccinated participants, and 20 pre-pandemic controls to measure the concentration of specific IgG antibodies against the SARS-CoV-2 spike protein, respectively.

**Results.:**

Vaccination with 3P+1M+1P induced a lower %CD3+ than 3P+1M; a lower %CD8+ than 3P and 3P+1M, but a higher %CD4+ than 3P+1M. All groups, whether vaccinated or not, presented a negative correlation in which a higher %CD8+ corresponded to a lower %CD4+. Cellular exhaustion in CD4+PD1+ of the 3P+1M group was higher than in all CD8+PD1+ of all vaccination platforms including the unvaccinated, except for 3P and the vaccinated or unvaccinated platforms in CD4+PD1+. No significant differences were found in IgG (anti-spike) production across vaccination platforms.

**Conclusions.:**

Homologous or heterologous vaccination platforms from three doses onwards do not modify IgG (anti-spike) antibody production; however, they improve CD8+ T cell values, which constitute the most important subpopulation for the antiviral response against SARS-CoV-2.

## INTRODUCTION

In December 2019, the first case of COVID-19 (coronavirus disease 2019), caused by SARS-CoV-2 (severe acute respiratory syndrome coronavirus 2), appeared in Wuhan, China ^1^^,^^2^. Its high transmissibility turned COVID-19 into a pandemic ^3^. By September 22, 2024, more than 776 million cases and 7.1 million deaths had been recorded worldwide ^(^^4^. In addition to biosafety measures, vaccination reduced cases of infection, hospitalization, and death ^5^. SARS-CoV-2 infection induces mucosal immunity in the upper respiratory tract. Innate immunity acts through PAMP receptors, which induce a response via type I and III interferons, which also link innate and adaptive immunity ^(^^6^. Cytotoxicity by natural killer (NK) cells together with adaptive immunity by B lymphocytes (LB) (immunoglobulin G, IgG and immunoglobulin A, IgA) and CD4+ and CD8+ (cluster of differentiation) T cells (TC) are activated to decrease viral load ^7^. Virus-specific CD4+ T cells are mostly Th1 (T helper) (IFN-g interferon gamma, IL-2 interleukin, IL-12p70) and not Th2 (IL-4 or IL-5); whereas CD8+ T cells are Th1 (IFN-g) and Th17 (IL-17) ^8^^-^^10^.

Vaccination and infection induce SARS-CoV-2 specific T cells that tolerate variant mutations better than antibodies, hence the interest in monitoring this population ^11^. On the other hand, CD8+ T cell cellular exhaustion appears in the presence of antigen persistence in viral infections or cancer and produces a failure in the development of memory CD8+ T cells and a deterioration in the functions of effector CD8+ T cells ^12^. In the acute and convalescent phase of COVID-19, cellular exhaustion is characterized by the gradual and progressive loss of cellular functions, sustained expression of inhibitory receptors or “checkpoints” (PD-1, programmed cell death protein 1; CTLA-4, cytotoxic T-lymphocyte-associated protein 4; NKG2A, NK cell lectin-like receptor subfamily C member 1; TIGIT, T cell immunoreceptor with Ig and ITIM domains; Tim-3, T-cell immunoglobulin and mucin-domain containing-3; VISTA, V-domain Ig suppressor of T-cell activation; CD73, CD244, Gal-9), changes in the epigenetic-transcriptional landscape, and metabolic reprogramming ^9^^,^^10^^,^^12^^-^^15^. In COVID-19 patients, high percentages of CD8+PD-1+ and CD4+PD-1+ T cells with high PD-1 levels, as well as CD8+ T cells and NK cells with high NKG2A levels, have been demonstrated, showing that SARS-CoV-2 induces cellular exhaustion ^14^^,^^16^^,^^17^. In COVID-19, there is lymphopenia and loss of total lymphocyte functionality ^7^^,^^14^^,^^16^^,^^18^. Diao *et al.* reported that 82.1% of patients with COVID-19 had lymphopenia, and furthermore that 522 COVID-19 cases had high production of PD-1 and Tim-3 in CD4+ and CD8+ T cells ^(^^16^. Therefore, recovering the quantity and function of total lymphocytes in patients with COVID-19 is fundamental. The global situation of this disease required the development and promotion of vaccines capable of stimulating robust immune responses against circulating SARS-CoV-2 variants ^7^^,^^19^.

In addition to effectiveness, the goal of vaccination is to reduce hospitalization, severe disease, and mortality ^5^. Thus, there are vaccines that have undergone randomized controlled trials such as Sinopharm (BBIBP-CorV), CoVaxin (BBV152), and Sinovac (CoronaVac) (inactivated vaccines); Janssen (Ad26.CoV2.S) and AstraZeneca (ChAdOx) (viral vector vaccines); Novavax (NVx-CoV2373) and Clover Biopharmaceuticals (SCD-2019) (protein subunit vaccines); and Pfizer-BioNTech (BNT162b2) and Moderna (mRNA-1273) (messenger RNA vaccines), demonstrating good efficacy, including against variants such as alpha, beta, gamma, and delta ^(^^20^. Vaccination began in Peru in February 2021 ^21^. Therefore, it is of interest to investigate not only the clinical or epidemiological effects of the different vaccination platforms but also the effects on the main immunological mechanisms that could be influenced. Studies reveal that, in the T cell response to the AstraZeneca vaccine, polyfunctional Th1 CD4+ and CD8+ T cells were found. With inactivated virus vaccines, there is more CD4+ T cell response than CD8+. In the case of mRNA vaccines, Th1-type CD4+ and CD8+ T cell responses were detected after the first dose of Pfizer and maintained for several months, but intervals of 16 weeks between doses also produce a robust response ^8^^,^^19^^,^^22^^,^^23^. With the Moderna vaccine, a minimal CD8+ T cell response was also found. Circulating Tfh (follicular helper T cells) have been found, demonstrating the role of these cells in antibody production, isotype switching, somatic hypermutation, and B cell proliferation ^6^^,^^24^^,^^25^. The Moderna vaccine induced a strong neutralizing antibody response, even superior to that of Pfizer; while both produced a greater response than the vector vaccines (AstraZeneca and Janssen) ^26^. A study in Mongolia compared four vaccines, showing a low humoral response in the case of Sinopharm and Sputnik compared to AstraZeneca and Pfizer ^(^^25^.

 It has not been observed following vaccination whether CD4+ and CD8+ T cells undergo cellular exhaustion, except for a transcriptomic study in healthy subjects immunized with the CoronaVac vaccine where elevated exhaustion scores were found in some GNLY (granulysin) effector CD4+ T cell and GZMK (granzyme K) CD8+ T cell genes, respectively. Just as in natural SARS-CoV-2 infection where cellular exhaustion exists, there is a gap in knowledge regarding T cell exhaustion after SARS-CoV-2 vaccination in the Peruvian adult population ^(^^27^. This study included people who received four vaccination platforms, with the purpose of analyzing the percentage variations of T lymphocyte subpopulations, characterizing their cellular exhaustion profiles, and quantifying the anti-spike IgG antibody response.

**KEY MESSAGES**

Motivation for conducting the study. There is a lack of knowledge regarding immune cell exhaustion following vaccination, as this is a population affected by SARS-CoV-2 infection.

Main findings. In the vaccinated groups, except for the 3P schedule, CD8+ T cells showed lower exhaustion than CD4+ T cells from the 3P+1M schedule. Furthermore, CD4+ T cell exhaustion levels were negatively correlated with those of CD8+ T cells, in both vaccinated and unvaccinated individuals.

Public health implications. These results may be useful for understanding the behavior of the post-vaccination immune response with homologous or heterologous platforms.

## MATERIALS AND METHODS

### Study design

An observational and cross-sectional study was conducted. Participant recruitment was carried out through the social networks of the Virology Research Center of the Universidad San Martín de Porres, as well as through a flyer, an explanatory video of the study objectives, and a form where interested parties could register to participate. People who filled out the form were summoned; an information session was held, they signed an informed consent form, data were collected, and a peripheral blood sample was taken. The collection process was carried out between April 17 and May 8, 2023.

### Sample and sampling

Sampling was non-probabilistic by convenience and consisted of n1=60 and n2=71 samples for cellular and humoral evaluation, respectively. According to the groups formed, the sample sizes were distributed as follows: n1=45 and n2=42 belonged to participants vaccinated with different vaccination schedules, n1=15 and n2=9 were samples from unvaccinated individuals, and n2=20 samples were obtained as a pre-pandemic control group (brucellosis serum bank from 2006), which was only analyzed for antibody measurement.

Vaccinated participants (n1=45 and n2=42) were stratified into groups according to the vaccination platform they received: 3 doses of Pfizer (3P, n1=n2=13); 3P plus one dose of Moderna (3P+1M, n1=13 and n2=12); 3P+1M plus one dose of Pfizer (3P+1M+1P, n1=12 and n2=11); and two doses of Sinopharm plus one of Pfizer (2S+1P, n1=07 and n2=06).

### Sample collection and flow cytometry analysis

**Figure 1:**
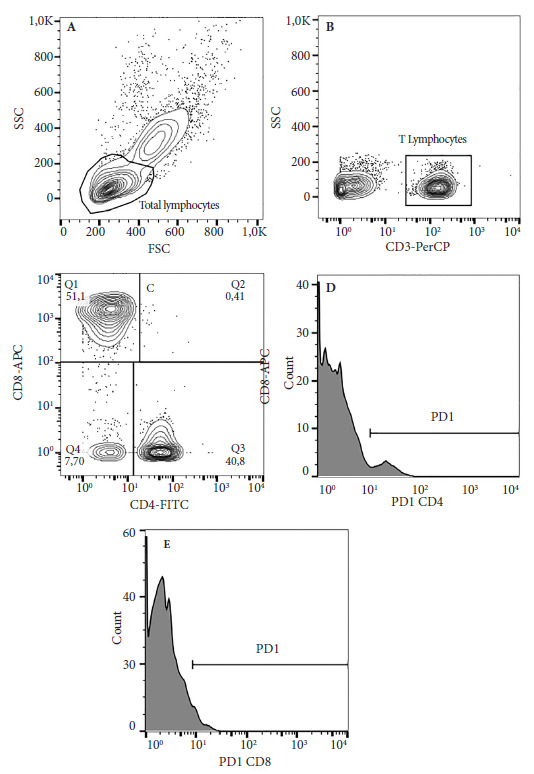
Strategy for flow cytometry analysis. 1x10⁶ PBMC/mL were obtained from individuals vaccinated or not against SARS-CoV-2, which were incubated for 24 h at 37°C and 5% CO2, stained with fluorescent antibodies against CD3, CD4, CD8, and PD-1, acquired on a FACSCalibur flow cytometer (BD Biosciences), and analyzed with FlowJo software. A. FSC vs. SSC showing total lymphocytes. B. SSC vs. CD3 showing T lymphocytes. C. CD4 vs. CD8 showing helper and cytotoxic T lymphocytes. D. Histogram showing the frequency of the PD-1 molecule in helper T lymphocytes. E. Histogram showing the frequency of the PD-1 molecule in cytotoxic T lymphocytes

FSC: Forward Scatter o dispersión frontral; SSC: Side Scatter o dispersión lateral; CD: Cluster of differentiation o grupo de diferenciación; PD-1: Programmed Death-1.

Peripheral blood samples were obtained in tubes with/without EDTA; peripheral blood mononuclear cells (PBMCs) were obtained by centrifugation gradient with Histopaque (SIGMA, USA) and cultured for 24 hours at 37 °C and 5% CO2 without any additional stimulus, and the sera were obtained by centrifugation and stored at -20 °C. Lymphocyte percentages and T cell exhaustion were detected using 100uL of PBMCs, which were stained with antibodies CD3-PerCp (Cat347344/SK7); CD4-FITC (Cat561842/RPA-T4); CD8-APC (Cat561953/RPA-T8) and PD1-PE (Cat557946/MIH4), all from BD Biosciences, USA. After staining, PBMCs were measured by flow cytometry in a FACSCalibur cellular analyzer (BD Biosciences) and data were analyzed with FlowJo v10. The analysis strategy consisted of determining the total lymphocyte population by size and granularity (Figure 1A); selecting the CD3+ T cell region with low granularity (Figure 1B); from the CD3+ cells, the CD8+CD4- (cytotoxic) and CD8-CD4+ (helper) subpopulations were determined in a dot plot using quadrant gating, respectively (Figure 1C); and from each subpopulation, using the histogram graph, the percentage levels and density of the PD-1 molecule were determined (Figures 1D and E).

### Enzyme-linked immunosorbent assay (ELISA) for IgG (anti-spike) determination

The commercial Epitope Diagnostic (EDITM) ELISA kit (Ref./KT-1032, USA) was used for the quantitative detection of anti-SARS-CoV-2 IgG antibodies against the Spike (S) protein in the serum of vaccinated and unvaccinated participants. In addition, a control group without antibodies for SARS-CoV-2 and with antibodies against *Brucella abortus* or pre-pandemic (n=10) was included. Assays were performed according to the manufacturer’s instructions. An ELx800 microplate spectrophotometric reader (BioTek Instruments Inc.) was used to read the optical density (OD) in all reactions. The threshold value for this kit was 60U/mL and data were analyzed with GraphPad Prism v9.0.

### Statistical analysis

Data distribution was evaluated using the Shapiro-Wilk test. Depending on compliance with the assumptions of normality and homogeneity of variances, the corresponding statistical tests were used. For data with normal distribution, one-way ANOVA was applied, followed by post hoc tests where pertinent. For non-normal data, non-parametric tests were used, such as Kruskal-Wallis and Dunn’s multiple comparison test. Correlations between continuous variables were analyzed using Pearson’s coefficient on parametric data. Continuous variables were expressed as median and 95% confidence interval, estimated using bootstrap percentiles. A p-value <0.05 was considered statistically significant. Analysis was performed with GraphPad Prism v9.0 software (GraphPad Software, Inc., San Diego, CA, USA).

### Ethical aspects

This study was approved by the Institutional Ethics Committee (CIE) in Research of the Universidad Peruana Cayetano Heredia, with approval code 416-35-22. Participation in the study was voluntary, following the signing of informed consent. Likewise, the ethical standards of the CIE and the basic international principles of justice, confidentiality, and autonomy necessary for research in humans were respected.

## RESULTS

### Percentage populations of total lymphocytes, T lymphocytes, and their CD4+ and CD8+ subpopulations in vaccinated or unvaccinated participants 

**Figure 2:**
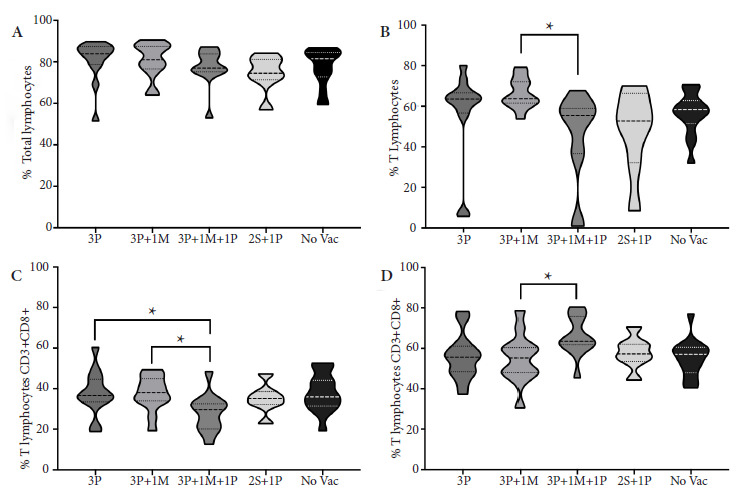
Vaccination schedules vs. lymphocyte population percentages. Lymphocyte percentages for each vaccination schedule were determined by flow cytometry. A. Total lymphocytes (FSC vs. SSC). B. CD3+ T lymphocytes. C. Cytotoxic T lymphocytes (CD3+CD8+) and D. Helper T lymphocytes (CD3+CD4+).

Multiple comparisons between vaccination groups were performed. The medians of the percentages of total lymphocytes (T, B, and NK) of all vaccination groups including the unvaccinated group showed broadly the same values (Figure 2A). Only the median percentage of T cells (CD3+) in the 3P+1M group (63.70%; 95% CI: 42.37-69.94) presented an increase (p<0.050) relative to the 3P+1M+1P group (55.40%; 95% CI: 30.97-59.55) (Figure 2B). The CD8+ subpopulations in the 3P (36.60%; 95% CI: 31.15-44.31) and 3P+1M (38.10%; 95% CI: 32.46-42.89) groups presented a higher median (p<0.050) than the 3P+1M+1P group (29.70%; 95% CI: 22.05-33.95) (Figure 2C); while the CD4+ in the group with 3P+1M (55.20%; 95% CI: 47.40-62.11) presented a lower median (p<0.050) than the 3P+1M+1P group (63.45%; 95% CI: 59.61-72.19) (Figure 2D). Regarding the 2S+1P group, in all cases, it behaved like the group of unvaccinated people.

3P: 3 doses of Pfizer; 3P+1M: 3 doses of Pfizer + 1 dose of Moderna; 3P+1M+1P: 3 doses of Pfizer + 1 dose of Moderna + 1 dose of Pfizer; 2S + 1 P: 2 doses of Sinopharm + 1 dose of Pfizer; No Vac: Unvaccinated. Kruskal-Wallis test (p<0.050), followed by a Dunn's multiple comparison test where Fig 2B and 2D schedules 3P+1M vs 3P+1M+1P (p=0.012 and p=0.049, respectively). Mann-Whitney test where Fig 2C schedules 3P vs 3P+1M+1P (p=0.009) and 3P+1M vs 3P+1M+1P (p=0.005), * statistical significance p < 0.050

### Correlation between T cell subpopulations according to vaccination platform

**Figure 3:**
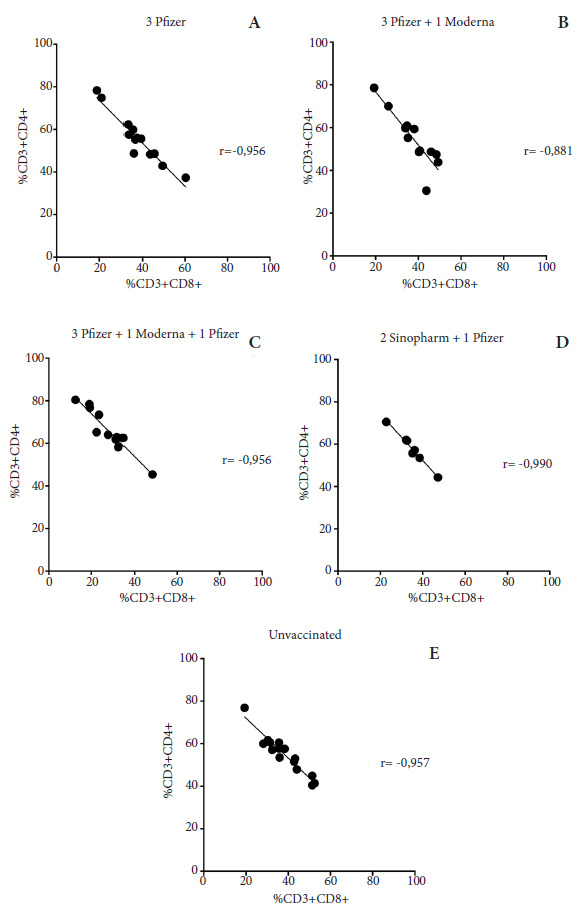
Correlation between T lymphocyte population percentages, CD3+CD4+, and CD3+CD8+ according to vaccination schedule. The percentage values of helper T lymphocytes (CD4+) and cytotoxic T lymphocytes (CD8+) were determined by flow cytometry. Assuming a Gaussian distribution, two-tailed Pearson's correlation was performed between these variables with a 95% confidence interval. Where r (rho) = correlation coefficient; negative r values close to -1 indicate a negative or inverse correlation.

In all groups of vaccinated or unvaccinated participants, a negative Pearson correlation was found between the percentages of CD3+CD4+ and CD3+CD8+ T cells; that is, the higher the percentage of cytotoxic T cells, the lower the percentage of helper T cells (Figure 3).

### Measurement of cellular exhaustion (PD-1+) in T cell subpopulations according to vaccination platform

**Figure 4:**
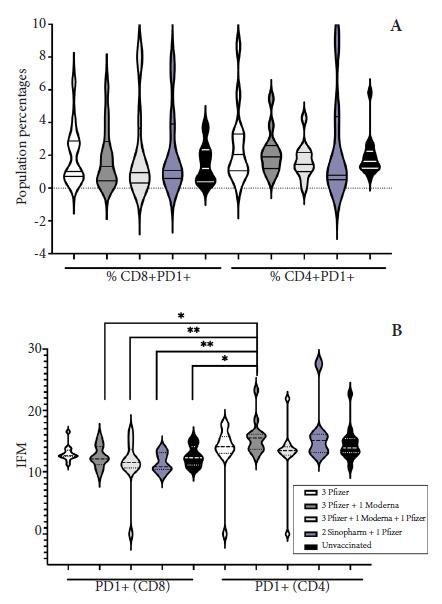
Percentages and MFI of PD-1+ T cells, CD8+ , and CD4+ according to vaccination schedule. Determined by flow cytometry: A. Percentages of PD-1+ cytotoxic and helper T cells. B. Cellular density of the PD-1+$ molecule (MFI) in cytotoxic and helper T cells. Mean Fluorescence Intensity (MFI).

The medians of the percentages of CD4+PD-1+ and CD8+PD-1+ T cells of the vaccinated and unvaccinated groups did not present significant differences (Figure 4A); but in the multiple comparison of these same groups and when analyzing the cellular density or mean fluorescence intensity (MFI) of PD-1 of both subpopulations, it was found that this molecule in the CD8+ T cells of all groups of vaccinated or unvaccinated individuals, except the 3P group, showed MFI median levels lower (p<0.050) than the CD4+ T cells of the 3P+1M group (Table 1) (Figure 4B).

P: 3 doses of Pfizer; 3P+1M: 3 doses of Pfizer + 1 dose of Moderna; 3P+1M+1P: 3 doses of Pfizer + 1 dose of Moderna + 1 dose of Pfizer; 2S + 1P: 2 doses of Sinopharm + 1 dose of Pfizer; No Vac = Not vaccinated.

**Table 1:** Cellular density of the PD-1 molecule (IFM) in LT, CD8^+^ and CD4^+^ according to vaccination scheme.

GROUP	Schedule	Mediana (MFI)	95% CI (MFI)	p-value
A	3Pfizer (PD1+CD8+)	12.60	12.19 - 13.74	>0.050
B	3Pfizer+1Moderna (PD1+CD8+)	12.10	11.41 - 13.58	0.038^a^
C	3Pfizer+1Moderna+1Pfizer (PD1+CD8+)	11.50	8.73 - 13.95	0.007^d^
D	2Sinopharm+1Pfizer (PD1+CD8+)	10.80	9.99 - 12.94	0.008^c^
E	Unvaccinated (PD1+CD8+)	12.30	11.59 - 13.43	0.023^b^
F	3Pfizer (PD1+CD4+)	14.10	10.90 - 16.22	>0.050
G	3Pfizer+1Moderna (PD1+CD4+)	15.50	13.92 - 17.30	>0.050
H	3Pfizer+1Moderna+1Pfizer (PD1+CD4+)	13.45	9.98 - 16.10	>0.050
I	2Sinopharm+1Pfizer (PD1+CD4+)	15.10	11.61 - 21.10	>0.050
J	Unvaccinated (PD1+CD4+)	13.90	12.97 - 15.99	>0.050

MFI=Mean Fluorescence Intensity; 95% CI=95% Confidence Interval. Kruskal-Wallis test (p<0.001), followed by a Dunn’s multiple comparison test where a B vs G, b E vs G and c D vs G (p<0.050). ANOVA test (p=0.008), followed by a Tukey’s multiple comparison test where d C vs G (p<0.040).

### IgG (anti-spike) antibody levels according to vaccination platform

**Figure 5:**
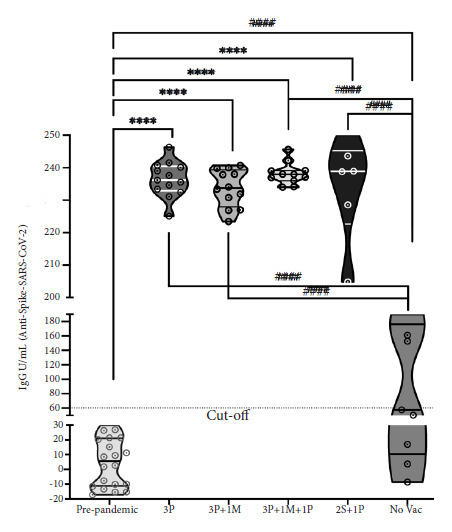
Levels of IgG (anti-spike) antibodies according to vaccination schedule. IgG antibody concentrations (U/mL}) in the serum of vaccinated or non-vaccinated individuals against SARS-CoV-2, including a pre-pandemic control group, were determined by enzyme immunoassay.

A group of sera obtained before December 2019, belonging to a brucellosis serum bank (year 2006), was used as a control group (n=10) without antibodies against SARS-CoV-2 (pre-pandemic). As expected, IgG (anti-spike) concentrations were found to be below the cutoff value (60U/mL) with a median of 5.58 U/mL (95% CI: 0-12.37). Furthermore, these concentrations were significantly lower (p<0.001) than all vaccination platforms including the unvaccinated group (Figure 5). In the case of the unvaccinated, the median was below the cutoff value (57.53 U/mL, 95% CI: 27.07-156.0) (Figure 5). In the comparison with the vaccination platforms, in all cases, there was a statistically significant difference (p<0.001) compared to the pre-pandemic control and the unvaccinated (Figure 5). In the multiple comparison between the vaccination platforms, no significant differences were found (p>0.050) and in this case, the median of these groups was 236.2 U/mL (95% CI: 233.2-239.7); 233.8 U/mL (95% CI: 230.0-237.3); 237.9 U/mL (95% CI: 235.9-240.5) and 238.9 U/mL (95% CI: 217.3-250.9) for 3P, 3P+1M, 3P+1M+1P and 2S+1P, respectively (Figure 5).

ut-off: assay threshold value equivalent to 60 U/mL; 3P: 3 doses of Pfizer; 3P+1M: 3 doses of Pfizer + 1 dose of Moderna; 3P+1M+1P: 3 doses of Pfizer + 1 dose of Moderna + 1 dose of Pfizer; 2S + 1P: 2 doses of Sinopharm + 1 dose of Pfizer; No Vac: not vaccinated. One-way ANOVA and Tukey's multiple comparison test. The violin plots represent the median and the limits are the 25th and 75th percentiles. Statistical significance if p < 0.0001. The **** indicate comparisons of the vaccination schedules vs. the pre-pandemic controls. The #### indicate comparisons of the vaccination schedules vs. the non-vaccinated group.

## DISCUSSION

The establishment of different vaccination platforms has helped reduce hospitalizations, disease severity, and mortality ^5^; however, it is necessary to generate evidence on the immune system components involved in vaccinated individuals.

According to Atmar *et al*., the vaccination strategy combining an mRNA vaccine like Pfizer and an adenovirus-vectored vaccine like AstraZeneca (heterologous platform) is more immunogenic than a homologous booster ).^28^ In our study, both heterologous and homologous platforms induce and do not modify the level of IgG (anti-spike) antibodies, similar to Atmar *et al*., 2022, who also demonstrated that second homologous or heterologous boosters with the Moderna vaccine produce similar IgG levels, even in antibody kinetics studies from 3 to 6 months ^29^. However, we found an improvement in the cellular response through the increase of T cell subpopulations and lower cellular exhaustion of CD8+ T cells, which may be associated with a longer duration of immunity. Kumar *et al*. found that between 28 days to 6 months post-vaccination, T cell subpopulations were similar. Atmar *et al*. determined that in 15 days, heterologous platforms (Moderna, Janssen, and Pfizer) induced a higher percentage of CD8+ T cells over CD4+ T cells ^28^. In our study, we found that homologous (3P) and heterologous (3P+1M) vaccination after 5.8 months (95% CI: 5.2-6.9) and 12.2 months (95% CI: 11.6-12.9) of the last dose correlate negatively, where a higher percentage of CD4+ T cells corresponds to a lower percentage of CD8+ T cells in peripheral blood. Although both subpopulations patrol secondary lymphoid organs and one would expect that in an antiviral response cytotoxic T cells should be in greater proportion, the presence of helper T cells remains important. In our results, vaccination favored the proportion of helper CD4+ T cells, which would remain as sentinel cells; while CD8+ T cells could be cells migrating from the circulation towards secondary lymphoid organs, becoming central and effector memory lymphocytes, a characteristic generated by vaccines, but it would also be interesting to evaluate the expression of homing receptors to corroborate this hypothesis ^30^. At the time of sampling, it was not possible to determine if any of the participants had active infection, which is a limitation of the study; therefore, the results found in this study must be taken in the context of an immune response induced by some possible contact, not evaluated, with the virus or by vaccination.

In the evaluation of the cellular immune response, the ideal would be the measurement of antigen-specific (anti-SARS-CoV-2) T cells, but this is not easy because it requires HLA (Human Leukocyte Antigen) typing and the determination of the antigenic epitope to detect a T cell ^31^. Although cellular exhaustion was first described in cancer, Diao *et al*. were among the first to describe this process in COVID-19, despite the existence of researchers like Shahbaz et al. who refute the concept of pathological cellular exhaustion of T cells, as they relate the increase of inhibitory receptors, such as PD-1, to the increase of cytokines by T cells ^10^. Our results agree with the concept of Diao *et al*. in the sense that as long as inhibitory receptors like PD-1 do not increase, T cells will be fit to mount an efficient cellular response against SARS-CoV-2.

The strength of this study is that it includes different vaccination schedules according to the reality of vaccination in Peru and also the use of control groups without antibodies and unvaccinated persons. Likewise, humoral and cellular responses were analyzed before and after the third vaccination dose, considering only the values obtained at that time point. The limitations of this study include a small sample size, which was chosen by convenience due to the difficulty in obtaining voluntary participation from donors; the stratification or heterogeneity of the vaccination schedules, because at the time of the study vaccinated individuals presented a higher proportion of these schedules; and the non-evaluation of antigen-specific T lymphocytes, because the use of this methodology is very expensive and requires more execution time per participant; however, these limitations do not influence the relevance of the findings.

In summary, we identified that homologous and heterologous platforms in three vaccination doses do not modify the humoral response and improve the T lymphocyte response in vaccinated individuals. These results also justify the need for booster vaccine doses, in accordance with the guidelines of local authorities, and emphasize the importance of evaluating B and T lymphocyte responses in vaccinated individuals.

In conclusion, homologous or heterologous vaccination platforms from three doses onwards do not modify the production of IgG (anti-spike) antibodies; however, they decrease the cellular exhaustion of CD8+ T cells relative to CD4+ and increase the percentage of CD4+ T cells, which are the most important subpopulation for the antiviral response against SARS-CoV-2.
