# Supplementary material for: T-cell exhaustion and antibody response in SARS-CoV-2 vaccine recipients
Source: Rev Peru Med Exp Salud Publica. 2025 Dec 1;42(4):370–8. doi: 10.17843/rpmesp.2025.424.14687 (PMC12879979; doi:10.17843/rpmesp.2025.424.14687)
Supplement: Translation

# Translation: Agotamiento de linfocitos T y respuesta de anticuerpos en vacunados contra SARS-CoV-2

**Authors:** Iván Lozada-Requena, Camila A. Arones-Santayana, Angela Vidal Riva, Joel de León, Arturo Pareja Cruz

**Affiliations:** 1 Laboratorio de Inmunología, Departamento de Ciencias Celulares y Moleculares, Facultad de Ciencias e Ingeniería, Universidad Peruana Cayetano Heredia, Lima, Perú.; 2 Centro de Investigación de Virología, Universidad de San Martín de Porres, Lima, Perú.

**Keywords:** Agotamiento Celular, IgG, TNF-a, Linfocitos T, PD-1, Vacuna

## Abstract

**Objetivos.:**

Evaluar las variaciones porcentuales de la población total de linfocitos, de los linfocitos T (LT) y de sus subpoblaciones; caracterizar los perfiles de agotamiento celular en LT CD4+ y CD8+; y cuantificar los niveles de anticuerpos IgG anti-spike en individuos que recibieron distintos esquemas de vacunación.

**Materiales y métodos.:**

Aislamos PBMC (células mononucleares de sangre periférica) de 45 participantes vacunados y 15 no vacunados para medir por citometría de flujo, porcentajes e IFM (intensidad de fluorescencia media) de LT. Además, obtuvimos suero de 42 participantes vacunados, 9 no vacunados y 20 controles prepandemia para medir la concentración de anticuerpos IgG específicos contra la proteína de la espícula del SARS-CoV-2, respectivamente.

**Resultados.:**

La vacunación con 3P+1M+1P indujo menor %CD3+ que 3P+1M; menor %CD8+ que 3P y 3P+1M, pero mayor %CD4+ que 3P+1M. Todos los grupos vacunados o no presentaron una correlación negativa en la que a mayor %CD8+ menor %CD4+. El agotamiento celular en CD4+PD1+ de 3P+1M fue mayor que en todos los CD8+PD1+ de todas las plataformas de vacunación incluyendo los no vacunados, excepto 3P y las plataformas de vacunados o no en CD4+PD1+. No se encontraron diferencias significativas en la producción de IgG (anti-spike) en las plataformas de vacunación.

**Conclusiones.:**

Las plataformas de vacunación homólogas o heterólogas a partir de tres dosis no modifican la producción de anticuerpos IgG (anti-spike); sin embargo, mejoran los valores de LTCD8+, que constituyen la subpoblación más importante para la respuesta antiviral contra el SARS-CoV-2.

## INTRODUCCIÓN

En diciembre del 2019 apareció el primer caso de COVID-19 (del inglés coronavirus disease 2019), por SARS-CoV-2 (del inglés severe acute respiratory syndrome coronavirus 2) en Wuhan, China ^1^^,^^2^. Su alta transmisibilidad convirtió al COVID-19 en una pandemia ^3^. Hasta el 22 de setiembre del 2024 se registraron más de 776 millones de casos y 7,1 millones de muertes a nivel mundial ^4^. Además de las medidas de bioseguridad, la vacunación redujo los casos de infección, hospitalización y muerte ^5^. La infección por SARS-CoV-2 induce una inmunidad de mucosas en el tracto respiratorio superior. La inmunidad innata actúa con receptores PAMP, que inducen una respuesta por interferones tipo I y III, que además enlazan la inmunidad innata y adaptativa ^6^. La citotoxicidad por células asesinas naturales (NK) junto con la inmunidad adaptativa por linfocitos B (LB) (inmunoglobilna G, IgG e inmunoglobulina A, IgA) y linfocitos T (LT) CD4^+^ y CD8^+^ (del inglés cluster of differentiation) se activan para disminuir la carga viral ^7^. Los LTCD4^+^ específicos al virus son mayormente Th1 (del inglés T helper) (IFN-g interferon gama, IL-2 interleuquina, IL-12p70) y no Th2 (IL-4 o IL5); mientras que los LTCD8^+^ son Th1 (IFN-g) y Th17 (IL-17) ^8^^-^^10^.

La vacunación e infección inducen LT SARS-CoV-2 específicos que toleran, mejor que los anticuerpos, las mutaciones de las variantes, por lo que surge el interés por monitorear esta población ^11^. Por otro lado, el agotamiento celular de LTCD8^+^ aparece ante una persistencia de antígenos en infecciones virales o cáncer y produce falla en el desarrollo de LTCD8^+^ de memoria y un deterioro en las funciones de LTCD8^+^ efectores ^12^. En la fase aguda y convaleciente del COVID-19 el agotamiento celular se caracteriza por la pérdida gradual y progresiva de las funciones celulares, expresión sostenida de receptores inhibidores o «checkpoints» (PD-1, proteína de muerte celular programada 1; CTLA-4, CitoTóxica Linfocito T-asociado a la proteína 4; NKG2A, Receptor C-tipo de lectina 2 de las células NK; TIGIT, inmunoreceptor de células T con dominios Ig e ITIM; Tim-3, Receptor de la familia de proteínas inmunoglobulina y mucina de dominio de células T; VISTA, del inglés, V-domain Ig suppressor of T-cell activation; CD73, CD244, Gal-9), cambios en el panorama epigenético-transcripcional y reprogramación metabólica ^9^^,^^10^^,^^12^^-^^15^. En pacientes con COVID-19 se ha demostrado altos porcentajes de LT CD8^+^PD-1^+^ y CD4^+^PD-1^+^ con altos niveles de PD-1, así como, LT CD8^+^ y NK con altos niveles de NKG2A, lo que demuestra que SARS-CoV-2 induce el agotamiento celular ^14^^,^^16^^,^^17^. En COVID-19 existe linfopenia y perdida de funcionalidad de linfocitos totales ^7^^,^^14^^,^^16^^,^^18^. Diao *et al.* reportaron que el 82,1% de pacientes con COVID-19 tenían linfopenia, y además que 522 casos de COVID-19 tenían alta producción de PD-1 y Tim-3 en LT CD4^+^ y CD8^+ (^^16^. Por tanto, recuperar la cantidad y la función de linfocitos totales en pacientes con COVID-19 es fundamental. La situación mundial de esta enfermedad requirió del desarrollo y promoción de vacunas capaces de estimular robustas respuestas inmunes contra variantes circulantes del SARS-CoV-2 ^7^^,^^19^.

Además de la efectividad, el objetivo de la vacunación es reducir la hospitalización, las enfermedades graves y la mortalidad ^5^. Es así que existen vacunas que han pasado por ensayos controlados-aleatorizados como Sinopharm (BBIBP-CorV), CoVaxin (BBV152) y Sinovac (CoronaVac) (vacunas inactivadas); Janssen (Ad26.CoV2.S) y AstraZeneca (ChAdOx) (vacunas vectorizadas); Novavax (NVx-CoV2373) y Clover Biopharmaceuticals (SCD-2019) (vacunas de subunidades proteicas) y Pfizer-BioNTech (BNT162b2) y Moderna (mRNA-1273) (vacunas de ARN mensajero) y han demostrado buena eficacia, incluso contra variantes como alpha, beta, gamma y delta ^20^. Desde febrero del 2021, en Perú se inició la vacunación ^21^. Por tanto, es de interés investigar no sólo los efectos clínicos o epidemiológicos de las diferentes plataformas de vacunación sino también los efectos sobre los principales mecanismos inmunológicos que podrían ser influenciados. Los estudios revelan que, en la respuesta por LT ante la vacuna de AstraZeneca se encontraron LT CD4^+^ Th1 y CD8^+^ polifuncionales. Con las vacunas de virus inactivado ocurre más respuesta de LT CD4^+^ que CD8^+^. En el caso de las vacunas de ARNm se detectaron respuestas de LT CD4 de tipo Th1 y CD8^+^ luego de la primera dosis de Pfizer y se mantuvo por varios meses, pero además intervalos de 16 semanas entre dosis producen una robusta respuesta ^8^^,^^19^^,^^22^^,^^23^. Con la vacuna de Moderna también se encontró mínima respuesta de LT CD8^+^. Se han encontrado Tfh (LT helper foliculares) en circulación, lo que demuestra el rol de estas células en la producción de anticuerpos, cambio de isotipo, hipermutación somática y proliferación de los LB ^6^^,^^24^^,^^25^. La vacuna de Moderna indujo una fuerte respuesta de anticuerpos neutralizantes, incluso superior a la de Pfizer; mientras que ambas produjeron mayor respuesta que las vacunas vectorizadas (AstraZeneca y Janssen) ^26^. Un estudio en Mongolia comparo cuatro vacunas, las que mostraron baja respuesta humoral en el caso de Sinopharm y Sputnik en comparación a AstraZeneca y Pfizer ^(^^25^.

No se ha observado tras la vacunación si los LTCD4^+^ y LTCD8^+^ sufren agotamiento celular, excepto por un estudio transcriptómico en sujetos sanos inmunizados con la vacuna de CoronaVac donde se encontraron puntajes elevados de agotamiento en algunos genes de LT CD4^+^ y CD8^+^ efectores GNLY (granulisina) y GZMK (granzima K), respectivamente. Al igual que la infección natural por SARS-CoV-2 donde si existe agotamiento celular, hay un vacío del conocimiento sobre el agotamiento de LT tras la vacunación contra SARS-CoV-2 en población adulta peruana ^27^. En este estudio se incluyeron personas que recibieron cuatro plataformas de vacunación, con el propósito de analizar las variaciones porcentuales de las subpoblaciones de linfocitos T, caracterizar sus perfiles de agotamiento celular y cuantificar la respuesta de anticuerpos IgG anti-spike.

**MENSAJE CLAVE**

Motivación para realizar el estudio. Existe un desconocimiento sobre el agotamiento de células inmunes luego de la vacunación, ya que es una población afectada por la infección de SARS-CoV-2.

Principales hallazgos. En los grupos vacunados, excepto en el esquema 3P, los LTCD8+ mostraron menor agotamiento que los LTCD4+ del esquema 3P+1M. Además, los niveles de agotamiento de LTCD4+ se correlacionaron de manera negativa con los de LTCD8+, tanto en vacunados como en no vacunados.

Implicancias para la salud pública. Estos resultados pueden ser útiles para comprender el comportamiento de la respuesta inmune posvacunación con plataformas homólogas o heterólogas.

## MATERIALES Y MÉTODOS

### Diseño de estudio

Se realizó un estudio de tipo observacional y transversal. La captación de participantes se llevó a cabo mediante las redes sociales del Centro de Investigación en Virología de la Universidad San Martín de Porres, así como a través de un volante, un video explicativo de los objetivos del estudio y un formulario en el que los interesados podían registrarse para participar. Las personas que llenaron el formulario fueron citadas; se realizó una sesión informativa, firmaron un consentimiento informado, se recopilaron los datos y se tomó una muestra de sangre periférica. El proceso de recolección se llevó a cabo entre el 17 de abril y el 8 de mayo de 2023.

### Muestra y muestreo

El muestreo fue no probabilístico por conveniencia y estuvo compuesto por n1=60 y n2=71 muestras para la evaluación celular y humoral, respectivamente. Según los grupos formados los tamaños de muestra se distribuyeron de la siguiente manera: n1=45 y n2=42 pertenecían a participantes vacunados con diferentes esquemas de vacunación, n1=15 y n2=9 fueron muestras de no vacunados y n2=20 muestras fueron obtenidas como grupo control prepandemia (seroteca de brucelosis del año 2006), el cual solo se analizó para la medición de anticuerpos.

Los participantes vacunados (n1=45 y n2=42) se estratificaron en grupos según la plataforma de vacunación que recibieron: 3 dosis de Pfizer (3P, n1=n2=13); 3P más una dosis de Moderna (3P+1M, n1=13 y n2=12); 3P+1M más una dosis de Pfizer (3P+1M+1P, n1=12 y n2=11) y dos dosis de Sinopharm más una de Pfizer (2S+1P, n1=07 y n2=06).

### Obtención de muestras y análisis de citometría de flujo

Las muestras de sangre periférica fueron obtenidas en tubos con/sin EDTA, las células mononucleares de sangre periférica (PBMC) fueron obtenidas por gradiente de centrifugación con Histopaque (SIGMA, USA) y sembradas por 24 horas a 37 °C y 5% de CO_2_ sin ningún estímulo adicional y los sueros fueron obtenidos por centrifugación y almacenados a -20 °C. Los porcentajes linfocitarios y el agotamiento de LT fue detectado usando 100uL de PBMC, los que fueron coloreados con anticuerpos CD3-PerCp (Cat347344/SK7); CD4-FITC (Cat561842/RPA-T4); CD8-APC (Cat561953/RPA-T8) y PD1-PE (Cat557946/MIH4), todos de BD Biosciences, USA. Después de la tinción las PBMC fueron medidas por citometría de flujo en un analizador celular FACSCalibur (BD Biosciences) y los datos fueron analizados con FlowJo v10. La estrategia de análisis consistió en determinar por tamaño y granularidad a la población de linfocitos totales (Figura 1A); seleccionar la región de LT CD3^+^ con escasa granularidad (figura 1B); a partir de los CD3^+^ se determinó, en una gráfica de puntos mediante “gating” de cuadrantes, las subpoblaciones CD8+CD4- (citotóxicos) y CD8^-^CD4^+^ (ayudadores), respectivamente (figura 1C) y a partir de cada subpoblación, usando la gráfica de histograma, se determinaron los niveles porcentuales y de densidad de la molécula PD-1 (figuras 1D y E). 

**Figura 1:** Estrategia para el análisis de citometría de flujo. Se obtuvieron 1x10^6^ PBMC/mL de per-sonas vacunadas o no contra el SARS-CoV-2, las que fueron incubadas por 24 h a 37 °C y 5% CO_2_, marcadas con anticuerpos fluorescentes contra CD3, CD4, CD8 y PD-1, adquiridas en un citómetro de flujo FACSCalibur (BD biosciences) y analizadas con el software FlowJo. A. FSC vs. SSC mostrando los linfocitos totales. B. SSC vs CD3 mostrando los linfocitos T. C. CD4 vs. CD8 mostrando linfocitos T helper y citotóxicos. D. Histograma mostrando la frecuencia de la molécula PD-1 en LT helper. E. Histograma mostrando la frecuencia de la molécula PD-1 en LT citotóxicos

FSC: Forward Scatter o dispersión frontral; SSC: Side Scatter o dispersión lateral; CD: Cluster of differentiation o grupo de diferenciación; PD-1: Programmed Death-1 o Muerte programada - 1.

### Inmuno ensayo enzimático (ELISA) para la determinación de IgG (anti-spike)

Se utilizó el kit comercial de ELISA de Epitope Diagnostic (EDI^TM^) (Ref./KT-1032, USA) para la detección cuantitativa de anticuerpos IgG anti-SARS-CoV-2 contra la proteína Spike (S) en el suero de participantes vacunados y no vacunados. Además, se incluyó un grupo control sin anticuerpos para SARS-CoV-2 y con anticuerpos contra *Brucella abortus* o prepandémico (n=10). Los ensayos se realizaron según las instrucciones del fabricante. Un lector espectrofotométrico de microplacas ELx800 (BioTek Instruments Inc.) fue usado para leer la densidad óptica (DO) en todas las reacciones. El valor umbral para este kit fue de 60U/mL y los datos fueron analizados con GraphPad Prism v9.0.

### Análisis estadístico

La distribución de los datos se evaluó mediante la prueba de Shapiro-Wilk. Según el cumplimiento de los supuestos de normalidad y homogeneidad de varianzas, se emplearon las pruebas estadísticas correspondientes. Para datos con distribución normal se aplicó ANOVA de una vía, seguido de pruebas post hoc cuando fue pertinente. Para datos no normales se utilizaron pruebas no paramétricas, como Kruskal-Wallis y la prueba de comparaciones múltiples de Dunn. Las correlaciones entre variables continuas se analizaron mediante el coeficiente de Pearson en datos paramétricos. Las variables continuas se expresaron como mediana e intervalo de confianza al 95%, estimado mediante percentiles bootstrap. Se consideró un valor de p<0,05 como estadísticamente significativo. El análisis se realizó con el programa GraphPad Prism v9.0 (GraphPad Sofware, Inc., San Diego, CA, USA).

### Aspectos éticos

El presente estudio contó con la aprobación del Comité Institucional de Ética (CIE) en Investigación de la Universidad Peruana Cayetano Heredia, con código de aprobación 416-35-22. La participación en el estudio fue voluntaria, luego de firmar el consentimiento informado. Así mismo, se respetaron los estándares éticos del CIE y los principios internacionales básicos de justicia, confidencialidad y autonomía necesarias para la investigación en humanos.

## RESULTADOS

### Poblaciones porcentuales de linfocitos totales, linfocitos T y sus subpoblaciones CD4+ y CD8+ en participantes vacunados o no vacunados

Se realizo la comparación múltiple entre grupos de vacunación. Las medianas de los porcentajes de linfocitos totales (T, B y NK) de todos los grupos de vacunación incluyendo el grupo de no vacunados mostraron en líneas generales los mismos valores (figura 2A). Únicamente la mediana del porcentaje de LT (CD3+) en el grupo 3P+1M (63,70%; IC 95%: 42,37-69,94) presentó un aumento (p<0,050) en relación con el grupo 3P+1M+1P (55,40%; IC 95%: 30,97-59,55) (figura 2B). Las subpoblaciones de CD8+ en los grupos 3P (36,60%; IC 95%: 31,15-44,31) y 3P+1M (38,10%; IC 95%: 32,46-42,89) presentaron mayor mediana (p<0,050) que el grupo 3P+1M+1P (29,70%; IC 95%: 22,05-33,95) (figura 2C); mientras que los CD4+ en el grupo con 3P+1M (55,20%; IC 95%: 47,40-62,11) presentó menor mediana (p<0,050) que el grupo 3P+1M+1P (63,45%; IC 95%: 59,61-72,19) (figura 2D). En cuanto al grupo 2S+1P, en todos los casos, se comportó como el grupo de personas no vacunadas.

**Figura 2:** Esquemas de vacunación vs. porcentajes poblacionales linfocitarias. Se determinaron por citometría de flujo los porcentajes de linfocitos para cada esquema de vacunación. A. Linfocitos totales (FSC vs. SSC). B. LT CD3+. C. LT citotóxicos (CD3+CD8+) y D. LT ayudadores (CD3+CD4+)

3P: 3 dosis de Pfizer; 3P+1M: 3 dosis de Pfizer + 1 dosis de Moderna; 3P+1M+1P: 3 dosis de Pfizer + 1 dosis de Moderna + 1 dosis Pfizer; 2S + 1 P: 2 dosis de Sinopharm + 1 dosis de Pfizer; No Vac: No vacunados. Prueba de Kruskall-Walis (p<0,050), seguido de un test Dunn de comparaciones múltiples donde Fig 2B y 2D esquemas 3P+1M vs 3P+1M+1P (p=0,012 y p=0,049, respectivamente). Prueba de Mann Whitney donde Fig 2C esquemas 3P vs 3P+1M+1P (p=0,009) y 3P+1M vs 3P+1M+1P (p=0,005), * significancia estadística p < 0,050

### Correlación entre subpoblaciones de LT según plataforma de vacunación

En todos los grupos de participantes vacunados o no vacunados se encontró una correlación de Pearson negativa entre los porcentajes de LT CD3^+^CD4^+^ y CD3^+^CD8^+^, es decir, a mayor porcentaje de LT citotóxicos, menor porcentaje de LT ayudadores (figura 3).

**Figura 3:** Correlación entre porcentajes poblacionales de LT, CD3+CD4+ y CD3+CD8+ según esquema de vacunación. Se determinó por citometría de flujo los valores porcentuales de LT ayudadores (CD4+) y citotóxicos (CD8+). Asumiendo una distribución Gaussiana, se realizó la correlación de Pearsonde dos colas entre estas variables con un intervalo de confianza de 95%. Donde r (rho) = coeficiente de correlación; los valores de r negativos y cercanos a -1 indican una correlación negativa o inversa.

### Medición de agotamiento celular (PD-1^+^) en subpoblaciones de LT según plataforma de vacunación

Las medianas de los porcentajes de LT CD4^+^PD-1^+^ y CD8^+^PD-1^+^ de los grupos de vacunados y no vacunados no presentaron diferencias significativas (figura 4A); pero en la comparación múltiple de estos mismos grupos y cuando se analiza la densidad celular o intensidad de fluorescencia media (IFM) del PD-1 de ambas subpoblaciones se encontró que esta molécula en los LT CD8^+^ de todos los grupos de vacunados o no vacunados, excepto el grupo 3P mostraron niveles de mediana de IFM menores (p<0,050) a los LT CD4^+^ del grupo 3P+1M (tabla 1) (figura 4B).

**Figura 4:** Porcentajes e IFM de LT, CD8+ y CD4+ con PD-1+ según esquema de vacunación. Se determinó por citometría de flujo: A. Por-centajes de LT citotóxicos y ayudadores PD-1+. B. Densidad celular de la molécula PD-1+ (IFM) en LT citotóxicos y ayudadores. Intensidad de fluorescencia media (IFM).

3P: 3 dosis de Pfizer; 3P+1M: 3 dosis de Pfizer + 1 dosis de Moderna; 3P+1M+1P: 3 dosis de Pfizer + 1 dosis de Moderna + 1 dosis Pfizer; 2S + 1 P: 2 dosis de Sinopharm + 1 dosis de Pfizer; No Vac = No vacunados.

**Tabla 1:** Densidad celular de la molécula PD-1 (IFM) en LT, CD8^+^ y CD4^+^ según esquema de vacunación.

GRUPO	Esquema	Mediana (IFM)	IC95% (IFM)	Valor de p
A	3Pfizer (PD1+CD8+)	12,60	12,19 - 13,74	>0,050
B	3Pfizer+1Moderna (PD1+CD8+)	12,10	11,41 - 13,58	0,038^a^
C	3Pfizer+1Moderna+1Pfizer (PD1+CD8+)	11,50	8,73 - 13,95	0,007^d^
D	2Sinopharm+1Pfizer (PD1+CD8+)	10,80	9,99 - 12,94	0,008^c^
E	No Vacunados (PD1+CD8+)	12,30	11,59 - 13,43	0,023^b^
F	3Pfizer (PD1+CD4+)	14,10	10,90 - 16,22	>0,050
G	3Pfizer+1Moderna (PD1+CD4+)	15.50	13,92 - 17,30	>0,050
H	3Pfizer+1Moderna+1Pfizer (PD1+CD4+)	13,45	9,98 - 16,10	>0,050
I	2Sinopharm+1Pfizer (PD1+CD4+)	15,10	11,61 - 21,10	>0,050
J	No Vacunados (PD1+CD4+)	13,90	12,97 - 15,99	>0,050

IFM=Intensidad de fluorescencia media; IC95%=Intervalo de confianza al 95%. Prueba de Kruskall-Walis (p<0,001), seguido de un test Dunn de comparaciones múltiples donde ^a^ B vs G, ^b^ E vs G y ^c^ D vs G (p<0,050). Prueba de ANOVA (p=0,008), seguido de un test de Tukey de comparaciones múltiples donde d C vs G (p<0,040).

### Niveles de anticuerpos IgG (anti-spike) según plataforma de vacunación

Se utilizó como grupo control (n=10) sin anticuerpos contra SARS-CoV-2 (prepandemia) a un grupo de sueros obtenidos antes de diciembre de 2019, los cuales pertenecen a una seroteca de brucelosis (año 2006). Como era de esperarse las concentraciones de IgG (anti-spike) se encontraron por debajo del valor de corte (60U/mL) con una mediana de 5,58 U/mL (IC 95%: 0-12,37). Además, estas concentraciones fueron significativamente menores (p<0,001) que todas las plataformas de vacunación incluyendo el grupo de no vacunados (figura 5). En el caso de los no vacunados la mediana se encuentra por debajo del valor de corte (57,53 U/mL, IC 95%: 27,07-156,0) (figura 5). En la comparación con las plataformas de vacunación en todos los casos existió diferencia estadísticamente significativa (p<0,001) respecto al control prepandemia y a los no vacunados (figura 5). En la comparación múltiple entre las plataformas de vacunación no se encontraron diferencias significativas (p>0,050) y en este caso la mediana de estos grupos fue de 236,2 U/mL (IC 95%: 233,2-239,7); 233,8 U/mL (IC 95%: 230,0-237,3); 237,9 U/mL (IC 95%: 235,9-240,5) y 238,9 U/mL (IC 95%: 217,3-250,9) para 3P, 3P+1M, 3P+1M+1P y 2S+1P, respectivamente (figura 5).

**Figura 5:** Niveles de anticuerpos IgG (anti-spike) según esquema de vacu-nación. Se determinó por enzima inmunoensayo las concentraciones de anticuerpos IgG (U/mL) en el suero de personas vacunadas o no contra el SARS-CoV-2, incluyendo un grupo control prepandemia.

Cut-off: valor umbral del ensayo que equivale a 60 U/mL; 3P: 3 dosis de Pfizer; 3P+1M: 3 dosis de Pfizer + 1 dosis de Moderna; 3P+1M+1P: 3 dosis de Pfizer + 1 dosis de Moderna + 1 dosis Pfizer; 2S + 1 P: 2 dosis de Sinopharm + 1 dosis de Pfizer; No Vac: no vacuna-dos. ANOVA one-way y prueba de comparaciones múltiples de Tukey. Las gráficas de violín representan la mediana y los límites los percentiles al 25% y 75%. Significancia estadística si ****/#### p < 0,0001. Los **** son las comparaciones de los esquemas de vacunación vs. los controles prepandemia. Los #### son las comparaciones de los esquemas de vacunación vs. los no vacunados.

## DISCUSIÓN

El establecimiento de distintas plataformas de vacunación ha ayudado a reducir las hospitalizaciones, la severidad de la enfermedad y la mortalidad ^5^; sin embargo, es necesario generar evidencias sobre los componentes del sistema inmune implicados en los vacunados.

Según Atmar *et al*. la estrategia de vacunación que combina una vacuna de RNAm como Pfizer y una vacuna vectorizada con adenovirus como AstraZeneca (plataforma heteróloga) es más inmunogénica que un refuerzo homólogo ^28^. En nuestro estudio tanto las plataformas heterólogas como las homólogas inducen y no modifican el nivel de anticuerpos IgG (anti-spike), similar a Atmar *et al,* 2022 quien además demuestra que los segundos refuerzos homólogos o heterólogos con la vacuna de Moderna producen similares niveles de IgG, incluso en estudios de cinética de anticuerpos de 3 a 6 meses ^29^. Sin embargo, encontramos una mejora en la respuesta celular a través del incremento de las subpoblaciones de LT y menor agotamiento celular de LTCD8^+^, lo que puede asociarse con mayor duración de la inmunidad. Kumar *et al.*, encontraron que entre 28 días hasta 6 meses posvacunación las subpoblaciones de LT fueron similares. Atmar *et al.* determinaron que en 15 días las plataformas heterólogas (Moderna, Jansen y Pfizer) indujeron un mayor porcentaje de LTCD8^+^ sobre los LTCD4^+(^^28^. En nuestro estudio encontramos que la vacunación homóloga (3P) y la heteróloga (3P+1M) luego de 5,8 meses (IC 95%: 5,2-6,9) y 12,2 meses (IC 95%: 11,6-12,9) de la última dosis se correlacionan negativamente donde a mayor porcentaje de LTCD4^+^ menor porcentaje de LTCD8^+^ en sangre periférica. Si bien ambas subpoblaciones patrullan los órganos linfoides secundarios y se esperaría que ante una respuesta antiviral los LT citotóxicos deberían estar en mayor proporción nunca deja de ser importante la presencia de LT ayudadores. En nuestros resultados la vacunación favoreció la proporción de LT CD4^+^ ayudadores, los que se mantendrían como células vigilantes; mientras que los LT CD8^+^ podrían ser células migrantes de la circulación hacia órganos linfoides secundarios, convirtiéndose en linfocitos de memoria central y efectora una característica que generan las vacunas, pero además sería interesante evaluar la expresión de receptores «homing» para corroborar esta hipótesis ^30^. Al momento de la toma de muestras, a ninguno de los participantes fue posible determinar si tenía infección activa, lo que resulta una limitación del estudio, por lo que los resultados encontrados en este estudio se deben tomar en un contexto de una respuesta inmune inducida por algún posible contacto, no evaluado, con el virus o por la vacunación.

En la evaluación de la respuesta inmune celular, lo ideal sería la medición de LT antígeno específicos (anti-SARS-CoV-2) pero esto no es fácil porque se requiere la tipificación de HLA (Antígeno Leucocitario Humano) y la determinación del epítopo antigénico para detectar a un LT ^31^. Si bien el agotamiento celular se describió por primera vez en cáncer fue Diao *et al*., uno de los primeros en describir este proceso en COVID-19 a pesar de que existen investigadores como Shahbaz *et al*. que refutan el concepto de agotamiento celular patológico de los LT, ya que relacionan el incremento de receptores inhibidores, como PD-1, con el aumento de citocinas por parte de LT ^10^. Nuestros resultados concuerdan con el concepto de Diao *et al.* en el sentido de que mientras receptores inhibidores como PD-1 no se incrementen los LT serán aptos para montar una eficiente respuesta celular contra el SARS-CoV-2.

La fortaleza de este estudio es que incluye distintos esquemas de vacunación según la realidad de la vacunación en Perú y además el uso de grupos control sin anticuerpos y de personas no vacunadas. Asimismo, se analizaron las respuestas humoral y celular antes y después de la tercera dosis de vacunación, considerando únicamente los valores obtenidos en ese punto temporal. Las limitaciones de este estudio incluyen un pequeño tamaño de muestra, el que fue elegido por conveniencia, debido a que fue difícil contar con la participación voluntaria de los donantes, la estratificación o heterogeneidad de los esquemas de vacunación, esto porque al momento del estudio los vacunados presentaban una mayor proporción de estos esquemas y la no evaluación de linfocitos T antígeno específicos, debido a que el uso de esta metodología es muy costosa y requiere mayor tiempo de ejecución por participante; sin embargo, estas limitaciones no influyen en la relevancia de los hallazgos.

En resumen, identificamos que las plataformas homólogas y heterólogas en tres dosis de vacunación no modifican la respuesta humoral y mejoran la respuesta de linfocitos T en los vacunados. Estos resultados también justifican la necesidad de dosis de vacunas de refuerzo, de acuerdo con los lineamientos de las autoridades locales y enfatiza la importancia de evaluar las respuestas de linfocitos B y T en los vacunados.

En conclusión, las plataformas de vacunación homólogas o heterólogas a partir de tres dosis no modifican la producción de anticuerpos IgG (anti-spike); sin embargo, disminuyen al agotamiento celular de LTCD8^+^ respecto a los CD4^+^ e incrementan el porcentaje de LTCD4^+^, los cuales son la subpoblación más importante para la respuesta antiviral contra el SARS-CoV-2.
